# Clinically significant prostate cancer detection and segmentation in low-risk patients using a convolutional neural network on multi-parametric MRI

**DOI:** 10.1007/s00330-020-07008-z

**Published:** 2020-06-27

**Authors:** Muhammad Arif, Ivo G. Schoots, Jose Castillo Tovar, Chris H. Bangma, Gabriel P. Krestin, Monique J. Roobol, Wiro Niessen, Jifke F. Veenland

**Affiliations:** 1 Department of Radiology & Nuclear Medicine, Erasmus University Medical Center, Wytemaweg 80, Room Na 2512 Erasmus MC, 3015 CN Rotterdam, The Netherlands; 2 Department of Urology, Erasmus University Medical Center, Rotterdam, The Netherlands

**Keywords:** Prostate cancer, Neural networks (computer), Active surveillance, Multi-parametric magnetic resonance imaging, Diagnosis, computer-assisted

## Abstract

**Objectives:**

To develop an automatic method for identification and segmentation of clinically significant prostate cancer in low-risk patients and to evaluate the performance in a routine clinical setting.

**Methods:**

A consecutive cohort (*n* = 292) from a prospective database of low-risk patients eligible for the active surveillance was selected. A 3-T multi-parametric MRI at 3 months after inclusion was performed. Histopathology from biopsies was used as reference standard. MRI positivity was defined as PI-RADS score ≥ 3, histopathology positivity was defined as ISUP grade ≥ 2. The selected cohort contained four patient groups: (1) MRI-positive targeted biopsy-positive (*n* = 116), (2) MRI-negative systematic biopsy-negative (*n* = 55), (3) MRI-positive targeted biopsy-negative (*n* = 113), (4) MRI-negative systematic biopsy-positive (*n* = 8). Group 1 was further divided into three sets and a 3D convolutional neural network was trained using different combinations of these sets. Two MRI sequences (T2w, *b* = 800 DWI) and the ADC map were used as separate input channels for the model. After training, the model was evaluated on the remaining group 1 patients together with the patients of groups 2 and 3 to identify and segment clinically significant prostate cancer.

**Results:**

The average sensitivity achieved was 82–92% at an average specificity of 43–76% with an area under the curve (AUC) of 0.65 to 0.89 for different lesion volumes ranging from > 0.03 to > 0.5 cc.

**Conclusions:**

The proposed deep learning computer-aided method yields promising results in identification and segmentation of clinically significant prostate cancer and in confirming low-risk cancer (ISUP grade ≤ 1) in patients on active surveillance.

**Key Points:**

*• Clinically significant prostate cancer identification and segmentation on multi-parametric MRI is feasible in low-risk patients using a deep neural network.*

*• The deep neural network for significant prostate cancer localization performs better for lesions with larger volumes sizes (> 0.5 cc) as compared to small lesions (> 0.03 cc).*

*• For the evaluation of automatic prostate cancer segmentation methods in the active surveillance cohort, the large discordance group (MRI positive, targeted biopsy negative) should be included.*

**Electronic supplementary material:**

The online version of this article (10.1007/s00330-020-07008-z) contains supplementary material, which is available to authorized users.

## Introduction

The standard clinical procedure for diagnosing prostate cancer (PCa) is a systematic transrectal ultrasound-guided (TRUS) biopsy, indicated by an elevated prostate-specific antigen (PSA) level and/or an abnormal digital rectal examination (DRE) [1]. However, this procedure results in low sensitivity and specificity [2, 3] leading to underdiagnosis of clinically significant PCa and overdiagnosis of insignificant PCa. Recently, multi-parametric magnetic resonance imaging (mpMRI) has been reported as a more accurate alternative for PCa characterization and detection [4–6]. A recent Cochrane review and meta-analysis has shown that mpMRI before prostate biopsy can aid in the selection of patients at risk of having clinically significant PCa [4]. In addition, MRI-targeted biopsy improves detection of significant PCa [5].

Radiologists use the Prostate Imaging Reporting and Data System (PI-RADS) v2 for visual lesion characterization on mpMRI [7]. PI-RADS v2 assessment uses a 5-point Likert scale ranging from 1 (highly unlikely to be malignant) to 5 (highly likely to be malignant) [7]. However, visual interpretation of mpMRI by radiologists suffers from large inter- and intra-observer variability [8]. Decreasing this variability is critical to improve PCa diagnosis and monitoring [9]. A computer-aided analysis of prostate mpMRI may improve PCa identification and may aid in standardization of MRI interpretation [10]. Ultimately, it may contribute in improving the diagnostic chain [11] and thereby reducing over- and underdiagnosis and treatment in prostate cancer management [10].

Different computer-aided methods [12–15] have been proposed to accurately identify PCa on mpMRI using a radiomics approach or deep learning network. The performance, quantified by the area under the receiving operating characteristic curve (AUC), ranges from 0.93 to 0.97 [14, 15]. The main limitation in these studies is that the selected patient cohorts consist of intermediate- and high-risk patients. These patients have primarily obvious and large (volume > 0.5 cc) lesions on MRI, and were mostly treated with a radical prostatectomy. There is no general agreement on the definition of clinically significant prostate cancer. According to PI-RADS v2, a clinically significant PCa should have histopathology ISUP grade ≥ 2 and/or volume ≥ 0.5 cc and/or have extra prostatic extension [7]. Most studies [12–14] excluded tumor volumes < 0.5 cc; therefore, these methods cannot be generalized to smaller volume PCa, which can be high grade and should be monitored in an active surveillance program. In daily diagnostic workup and MRI reading, the number of obvious cases is limited; moreover, these cases do not cause the substantial reading variability. Furthermore, the challenging cases with discordance between the PIRADS score and the histopathological findings were not included in these studies.

We hypothesize that the potential additional clinical value of MRI-based computer-aided method will be most substantial in low-risk patients who opt for active surveillance. Active surveillance is considered a treatment option for patients diagnosed with a clinically insignificant PCa [16, 17]. These low-risk patients most likely do not have high volume or clinically significant tumors; however, they may benefit from a timely diagnosis to prohibit tumor progression to a clinically significant PCa. Current active surveillance protocols require monitoring with regular clinical evaluations and prostate biopsies. The mpMRI is increasingly used to monitor non-invasively the low-risk PCa patients on active surveillance and to enable targeted biopsies [18–20]. Assistance in identification and segmentation of clinically significant PCa may reduce MRI-reading variability in active surveillance patients.

In this study, we aim to detect and segment clinically significant PCa in a prospective clinical cohort of low-risk patients on active surveillance using an MRI-based deep learning approach and evaluate its performance in a routine clinical setting.

## Materials and methods

### Patient cohort

The study was HIPAA compliant and written informed consent with guarantee of confidentiality was obtained from the participants. Initially, 377 patients with low-risk PCa (defined as International Society of Urological Pathology “ISUP,” grade 1) were prospectively enrolled in our in-house database from 2016 to 2019 as part of the global MRI-PRIAS protocol (www.prias-project.org), a web-based active surveillance study with defined criteria for inclusion and follow-up [21]. All participants received a multi-parametric MRI and targeted biopsies of visible suspicious (PI-RADS ≥ 3) lesions at baseline (3 months after diagnosis) and during every repeat standard TRUS-guided biopsy, scheduled at 1, 4, 7, and 10 years after diagnosis. A detailed description of the clinical workup was recently published [22].

**Fig. 1:**
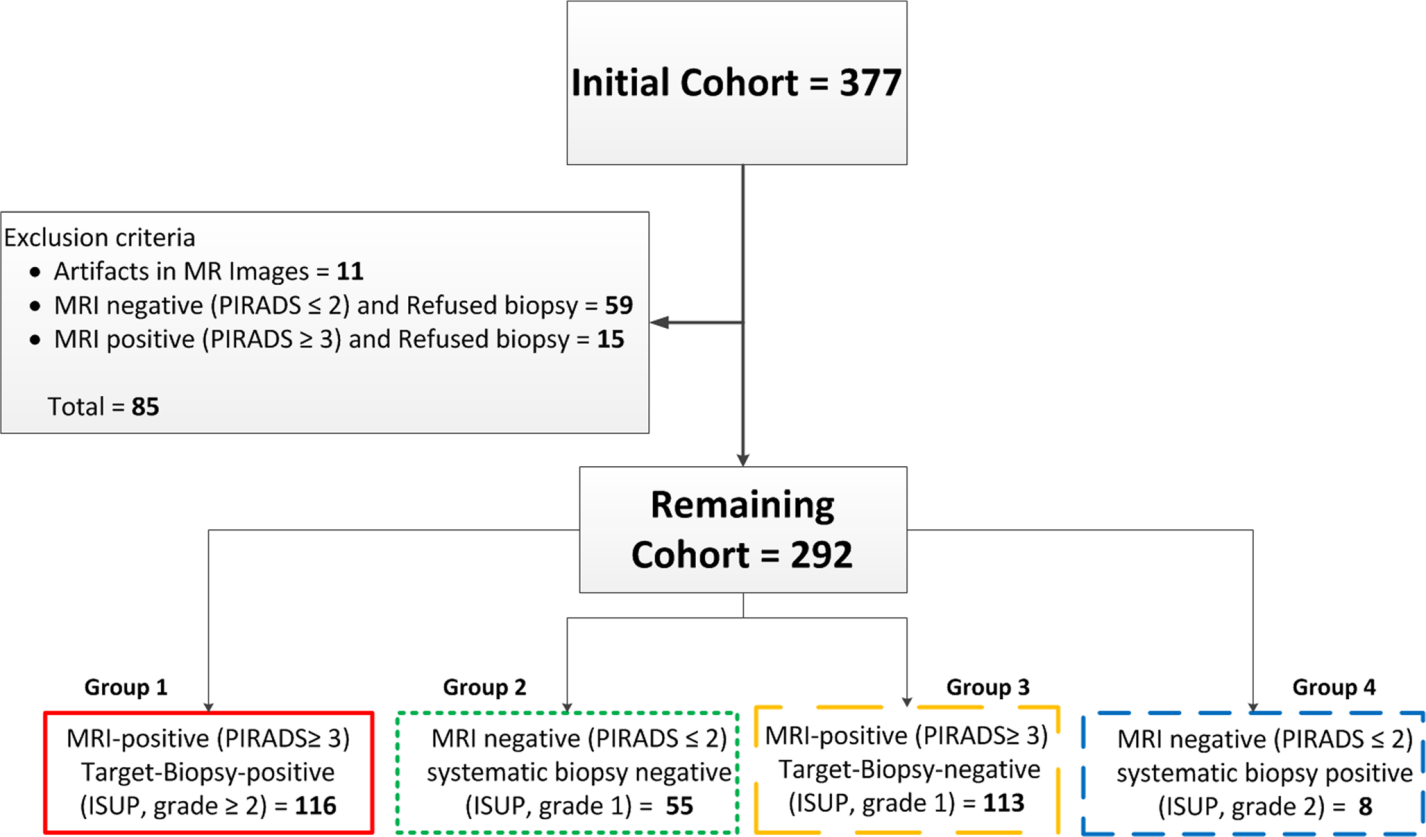
Flow diagram of patient’s exclusion and inclusion process in the study. ISUP, International Society of Urological Pathology; PI-RADS, Prostate Imaging Reporting and Data System

For each patient, two MRI sequences, i.e., a T2-weighted imaging (T2w) and a high *b*-value diffusion-weighted image (DWI) at *b* = 800, and the apparent diffusion coefficient (ADC) map were selected. Histopathology data from MRI-targeted biopsies were also extracted and considered reference standard. Patients who refused or had no biopsy procedure or whose MR images showed artifacts were excluded from the study (Fig. 1).

The remaining cohort (*n* = 292) was divided into four groups (Fig. 1) based on findings:

- Group 1. One hundred sixteen patients with positive lesions on MRI (PI-RADS score ≥ 3) who had positive targeted biopsies (ISUP grade ≥ 2)
- Group 2. Fifty-five patients with negative MRI (PI-RADS score ≤ 2) who had negative systematic biopsies (ISUP grade ≤ 1)
- Group 3. One hundred thirteen patients with positive lesions on MRI (PI-RADS score ≥ 3) who had negative targeted biopsies (ISUP grade ≤ 1)
- Group 4. Eight patients with negative MRI (PI-RADS score ≤ 2) who had positive systematic biopsies (ISUP grade ≥ 2)

Clinically non-significant and significant PCa were defined based on histopathology-defined ISUP grade or Gleason score [23].

- ISUP grade ≤ 1(Gleason score ≤ 3 + 3 = 6): uniform glands that look similar to normal cells and suggest low-risk PCa.
- ISUP grade = 2 (Gleason score 3 + 4 = 7): predominant uniform glands look similar to normal cells with less poorly formed glands which suggest intermediate-risk PCa.
- ISUP grade = 3 (Gleason score 4 + 3 = 7): predominant poorly formed glands which suggest intermediate-risk PCa with less uniform glands that look similar to normal cells.
- ISUP grade ≥ 4 (Gleason score ≥ 8): only poorly formed glands suggest high-risk PCa.

The patient characteristics, grouped based on the found ISUP grade, are listed in Table 1. The total number of lesions are divided in two zones (peripheral and transition) and also reported in the Table 1. A sub-cohort analysis of the transitional zone vs. peripheral zone was done and presented as supplementary material.

**Table 1:** Details of the patient cohort (*n* = 292) included in this study (median and interquartile range)

	ISUP grade = 1	ISUP grade = 2	ISUP grade = 3	ISUP grade = 4	ISUP grade = 5	ISUP grade = 1–5
Number of patients	155	110	18	4	5	292
Age	66 [60–72]	69 [64–72]	69 [63–73]	73 [59–77]	68 [64–72]	68 [62–72]
PSA	7.8 [5.7–11]	8.1 [6.2–12.2]	9.6 [7.7–12.7]	12.5 [7.6–17.5]	8.6 [5.9–15]	8.2 [5.9–12]
PSA density	0.18 [0.11–0.28]	0.22 [0.14–0.32]	0.25 [0.19–0.41]	0.24 [0.13–0.33]	0.15 [0.11–0.25]	0.19 [0.12–0.30]
Prostate volume (cc)	45 [31–66]	38 [30–58]	28 [23–44]	48 [37–98]	51 [32–130]	41 [30–61]
No. of lesion	–	1 [1–1]	1 [1–2]	1 [1–2]	1 [1–2]	1 [1–1]
Total lesion in PZ	–	92	12	3	5	112
Total lesions in TZ	–	19	3	0	1	23
Lesion volume (cc)	–	0.36 [0.19–0.78]	0.30 [0.21–1.25]	0.10 [0.09–6.12]	1.25 [0.28–1.90]	0.34 [0.18–0.82]
No. of positive targeted biopsies	–	3 [2–4]	3 [2–4]	2 [1–4]	2 [2–5]	3 [2–4]
PIRADS score	3 [2–4]	4 [4–5]	4 [4–5]	3 [3–5]	5 [4–5]	4 [3–4]

### Magnetic resonance imaging and pre-processing

The MRI protocol included T2-weighted imaging (T2w), diffusion-weighted imaging (DWI) from which apparent diffusion coefficient (ADC) maps were constructed, and dynamic contrast-enhanced (DCE) imaging, according to the PI-RADS v2 guidelines [7]. Detail of acquisition parameters are presented in the supplementary material (Table S3). MRI was performed on a 3-T system (Discovery MR750, GE Healthcare) using a 32-channel pelvic phased-array coil. All MRIs were reviewed by one urogenital radiologist with over 6 years of prostate MRI experience. Individual lesions were scored according to the PI-RADS v2 5-point Likert scale for high-grade PCa. Visible MRI lesions with a PI-RADS score from 3 to 5 were defined as suspicious and delineated. The fusion technique of MRI and TRUS was used (Koelis UroStation™) to perform targeted biopsies of all suspicious lesions, identified on MRI. The suspicious MRI lesions, delineated on DICOM images, were targeted with a maximum of four cores under ultrasound guidance. Experienced operators performed the biopsy procedures. One expert uropathologist reviewed biopsy specimens according to the ISUP 2014 modified Gleason Score [23].

For every patient in our cohort, suspicious lesions were evaluated according to PI-RADS v2 guidelines, with the DWI and ADC maps as the dominant sequence for peripheral zone lesions and T2W images for the transition zone lesions [7]. All manual delineations of suspected lesions were translated to T2w images using AW server 2.0 (GE Healthcare). Delineated T2w images are necessary in MRI/US fusion method to provide image guidance for targeted biopsy procedure, as T2w images contain more anatomical information as compared to DWI or ADC maps. The manual delineation of the suspicious lesion on T2w images was used for reference ground truth (binary mask) for each lesion having ISUP grade ≥ 2. For each patient, the DWI images with ADC values were manually rigidly co-registered to the T2w images. Moreover, the mpMRI images (T2w image, DWI (b800), and ADC) with reference ground truth were resampled to a uniform voxel spacing of 0.371 × 0.371 × 3.3 mm. Furthermore, the 3D images were cropped to the whole prostate region of interest having dimension 192 × 128 × 24 voxels along *x*, *y*, and *z* directions.

### Convolutional neural network

**Fig. 2:**
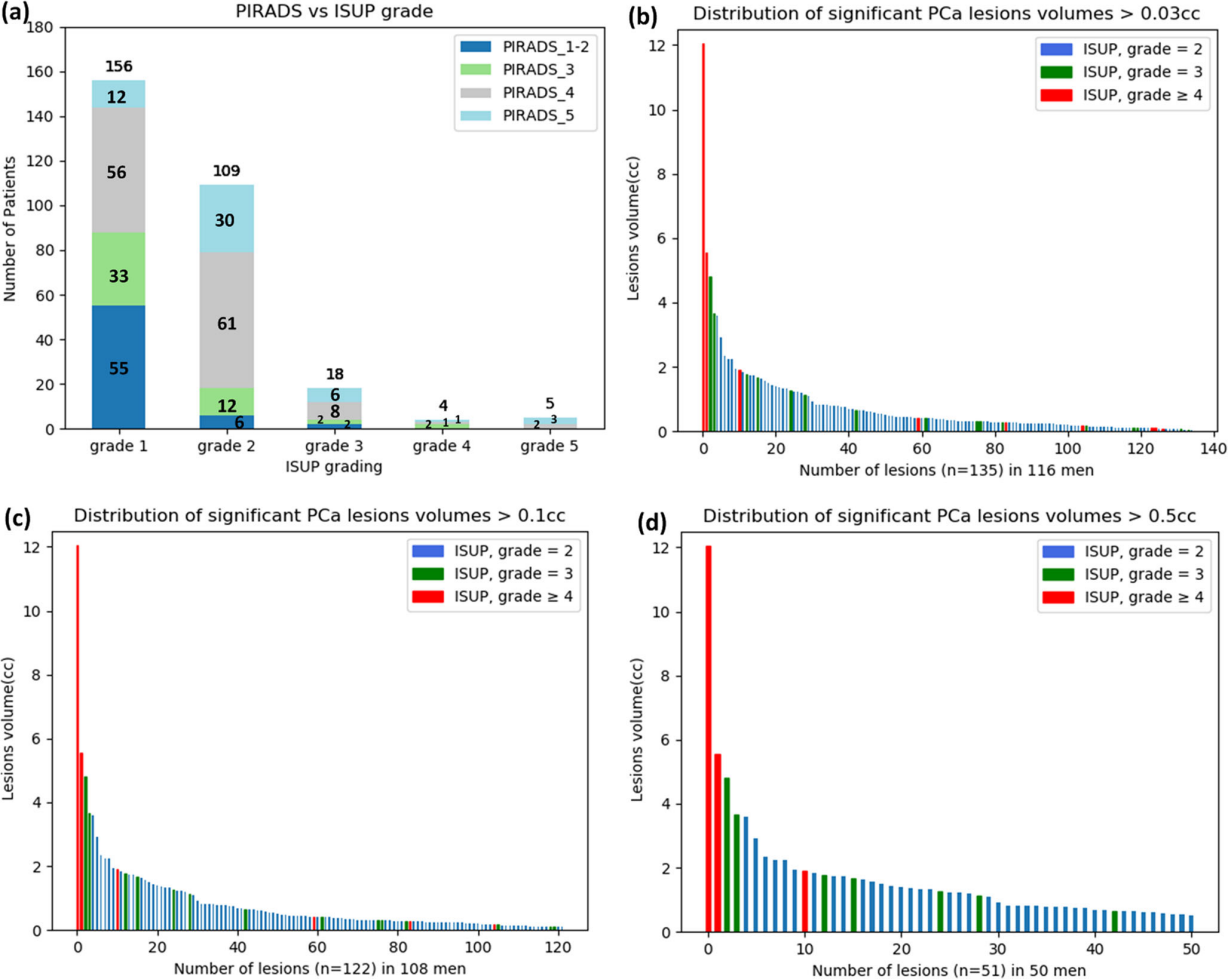
**a** The division of patients into different ISUP grades in direct relation to assigned PI-RADS v2 score. **b**–**d** Significant prostate cancer lesion volume distribution for different volumes ranging from > 0.03 to > 0.5 ml in patients on active surveillance. The volumes are presented, based on the ISUP grade. ISUP, International Society of Urological Pathology; PI-RADS, Prostate Imaging Reporting and Data System

The developed convolution neural network (CNN) [24] takes three MRI images; the T2-weighted (T2w) image, the diffusion-weighted image (DWI), and the apparent diffusion coefficient (ADC) maps as input and consider each sequence a separate input channel to generate a PCa segmentation (Fig. 2a).

The network contains twelve single 3D convolution layers with 3 × 3 × 3 kernel size, followed by a Rectified Linear Unit (ReLU). In the down-sampling and up-sampling blocks, at the last two layers of the network, a 3 × 3 × 1 kernel size filter was used due to the small image size in the *z*-axis. Batch normalization (BN) was added after each 3D convolution to improve convergence speed during training [25]. A concatenation with the corresponding computed featured map from the down-sampling part was performed after up-sampling. In the final layer, a 3D convolution having 1 × 1 × 1 kernel size was used to map computed features to the predicted PCa segmentation. In each convolution layer, appropriate padding was used. A schematic representation of the used CNN is shown in Fig. 2b.

The training of the network was implemented in Keras (version 2.0.2) with Tensor Flow (version 1.0.1) as backend in Python (version 3.5.3). The training and prediction was performed on a GeForce GTX TITAN Xp GPU (NVIDIA). The loss function during training was the binary cross-entropy metric and optimized using Adam optimizer [26] with a learning rate of 0.01. As the number of annotated data was limited, data augmentation was implemented; rotation (0–5^°^, along *x*,*y*,*z*-axes) and shearing (along *x*,*y*,*z*-axes) with rigid transformation and 50% probability for all images during training. This allows the network to learn invariance to such deformations and also helps to prevent overfitting and to generalize better. The total number of epochs was set to 500. The output of the trained network was a binary segmentation of clinically significant PCa lesions.

#### Prostate cancer segmentation

**Fig. 3:**
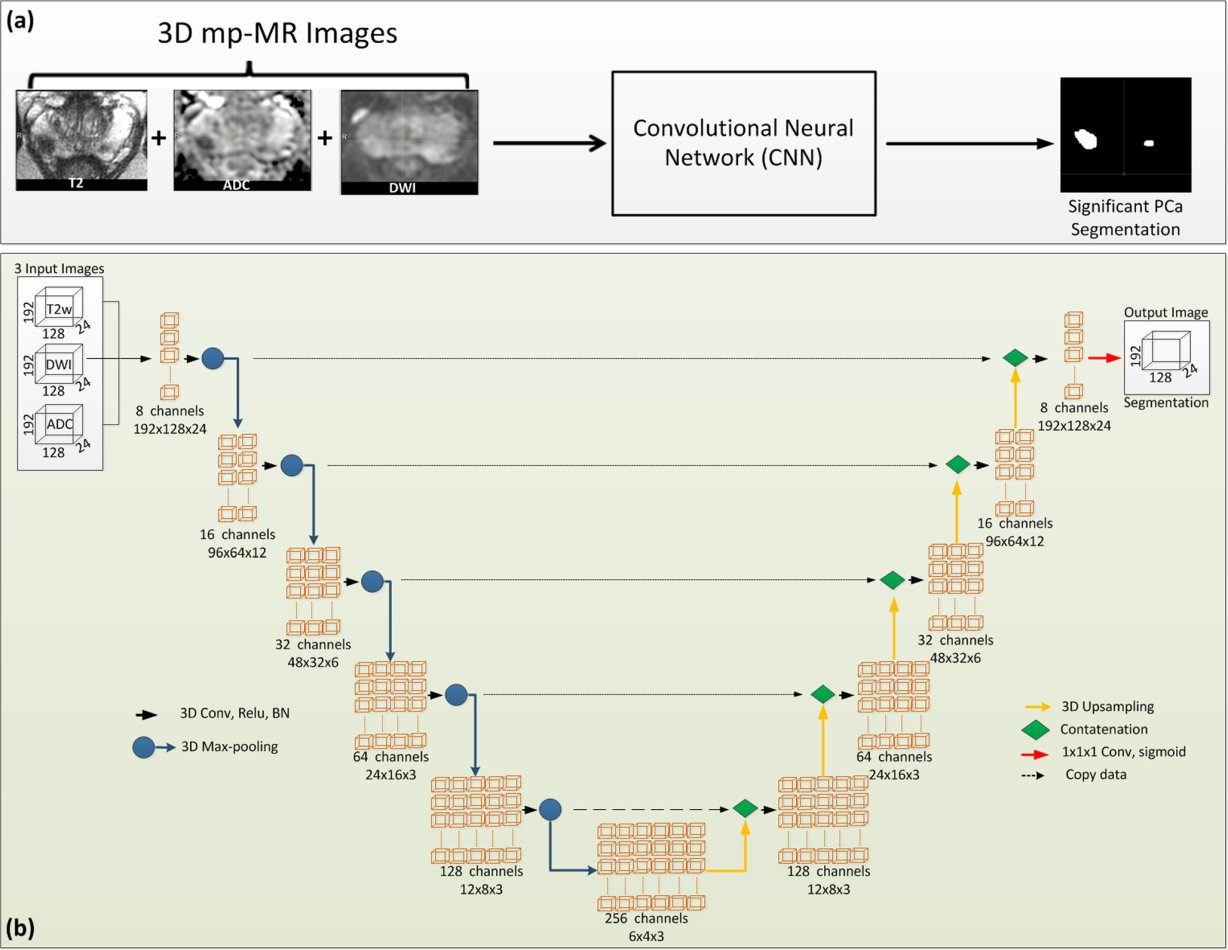
**a** Schematic diagram of the proposed method to segment significant PCa using a convolution neural network on mpMRI (T2w image, DWI b800, ADC map) as input and considering each image as a separate input channel. **b** Schematic representation of the convolutional neural network (CNN) architecture

In the experiments, the MRI-positive targeted biopsy-positive group (*n* = 116) was randomly divided into three sets (Fig. 3). The CNN model was trained as described in the “convolutional neural network” section, in threefold cross-validation using different combinations of these sets. The three trained networks were named model 1, model 2, and model 3. The MRI-negative systematic biopsy-negative group 2 (*n* = 55) was not used in the training because of the absence of PCa lesions in these images. Also, group 3 (i.e., patients with a positive MRI but negative targeted biopsy) was not included in the training set due to the absence of ISUP ≥ 2 grade prostate cancer. After training, each trained model was used to predict PCa on the corresponding test data. The systematic biopsy locations were not available; therefore, patients found with significant PCa based on systematic biopsies in group 3 and group 4 (*n* = 21) were excluded from testing (Fig. 3).

### Statistical analysis

To evaluate the performance of the method, the sensitivity and the specificity were calculated and receiver operating characteristic (ROC) curves were plotted for three different lesion volumes (0.03 cc, 0.1 cc, and 0.5 cc) of the segmented lesions. For each of the three lesion volumes, the sensitivity was calculated only for the patients with lesion volumes higher than the threshold volume. The lesion volume thresholds were selected based on the minimum significant PCa lesion volume (0.031 cc) in our data and the standard maximum lesion volume of clinically significant PCa based on the PI-RADS v2 definition. The lesions volume was calculated by multiplying the total number of voxels in the lesion with the voxel size (0.371 mm × 0.371 mm × 3.3 mm).The lesion segmentation was considered true positive when the overlap lesion volume between the reference ground truth and segmented lesion is larger than 0.01 cc.

## Results

### Patient cohort analysis

**Fig. 4:**
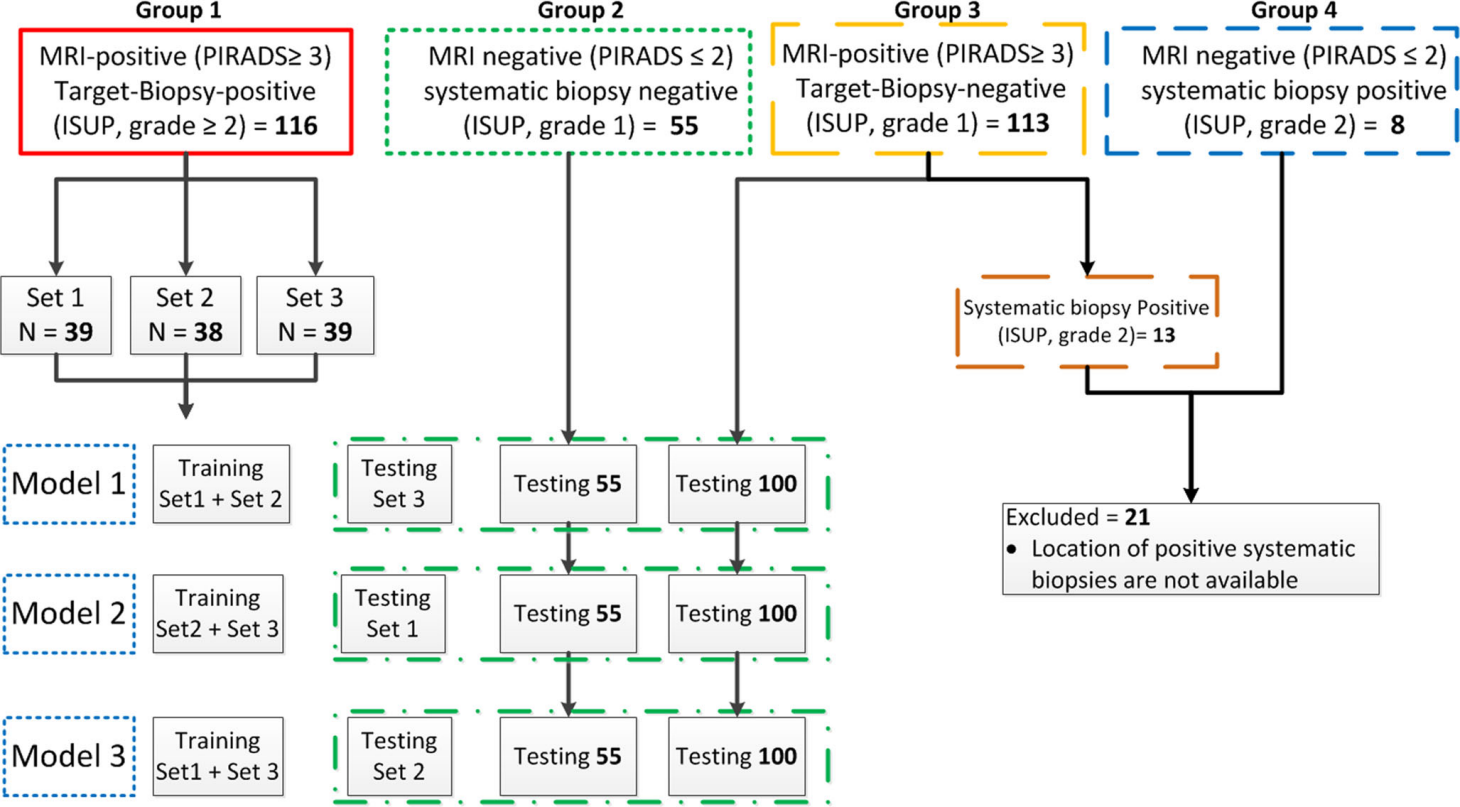
Flow diagram of patient’s selection and division into training and testing datasets. The group 1 patients (*n* = 116) with positive lesions on MRI (PI-RADS score ≥ 3) who had positive targeted biopsies (ISUP grade ≥ 2) was divided into three sets. The network was trained in threefold cross-validation combining two of these sets in all possible combinations. The evaluation was performed on the left-out positive set and the negative cases from group 2 (*n* = 55) and group 3 (*n* = 100). Since the systematic biopsy locations were not available, patients found with significant PCa based on systematic biopsies in group 3 (*n* = 13) and group 4 (*n* = 8) were excluded from training and testing

The division of patients into the different ISUP grade groups and their relation with the assigned PIRADS score (Fig. 4a) show that many patients (*n* = 101) were scored PI-RADS ≥ 3 by the radiologist but these patients had no significant PCa based on targeted biopsies (specificity = 33%). Also, some patients (*n* = 8) were assigned PI-RADS score ≤ 2 and had significant PCa based on the systematic biopsies procedure (sensitivity = 94%). The lesion volume distribution (Fig. 4b, c, d) of the significant PCa from > 0.03 to > 0.5 cc showed that the study data contained a wide range of lesion volumes (0.031–12.06 cc) and approximately 81% of them have ISUP grade = 2.

### Prostate cancer segmentation

**Fig. 5:**
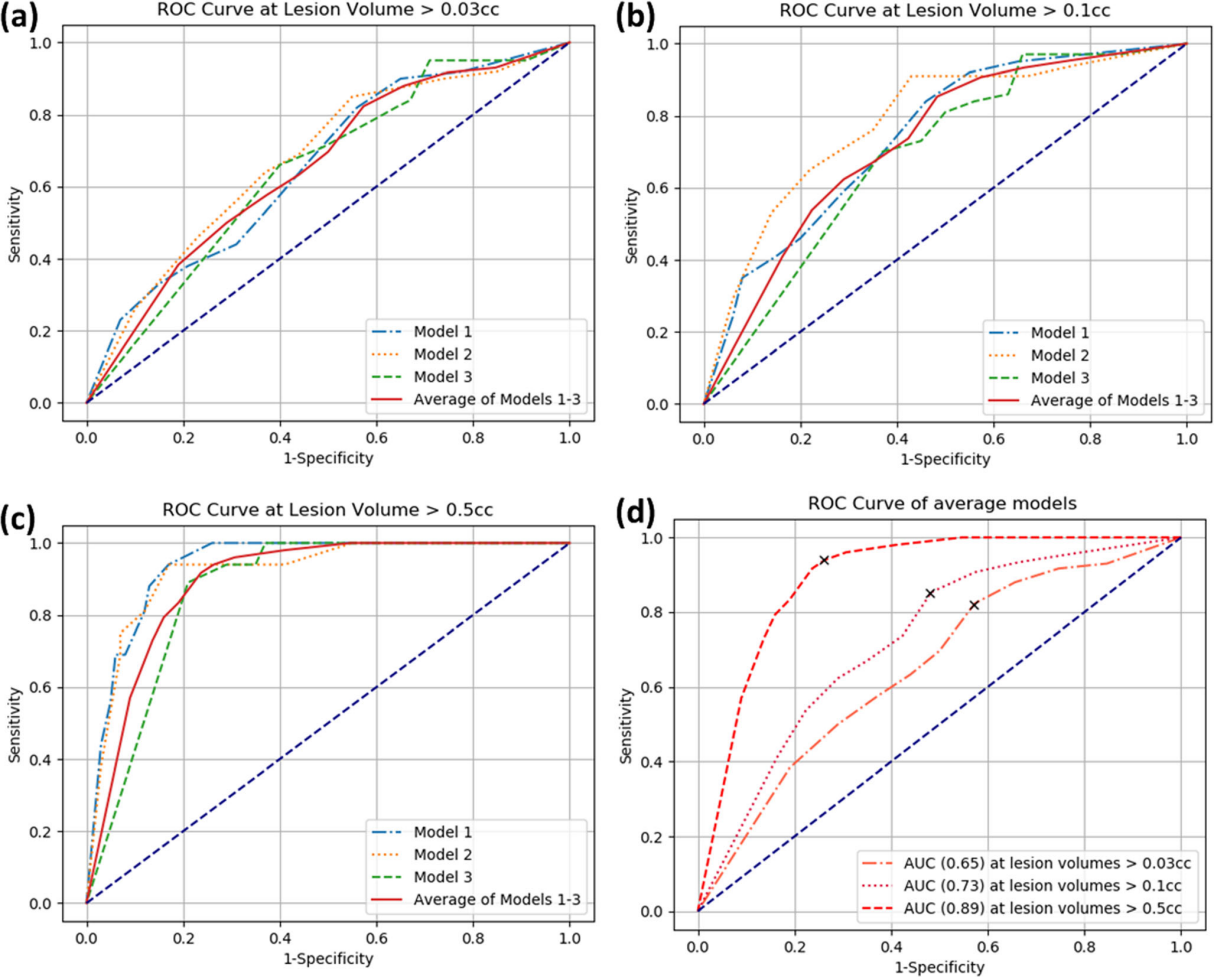
The ROC curves of the three models generated on the test set following threefold cross-validation and their average for different lesion volumes: (**a**) volume > 0.03 cc, (**b**) volume > 0.1 cc, (**c**) volume > 0.5 cc. The sensitivity and specificity computed at the best cutoff point are indicated. ROC, receiver operator characteristics

For patients with tumor volumes > 0.03 cc (number of lesions = 135), the average sensitivity was 82% at an average specificity of 43% with AUC 0.65. For patients with tumor volume > 0.1 cc images (number of lesions = 123), the average sensitivity was 85% at an average specificity of 52% with AUC 0.73. It further improves to 94% sensitivity and 74% specificity with AUC 0.89 for patients with tumor volumes > 0.5 cc (number of lesions = 51). The cutoff points used to calculate above-stated sensitivity and specificity for the three ROC curves are shown in Fig. 5d.

**Fig. 6:**
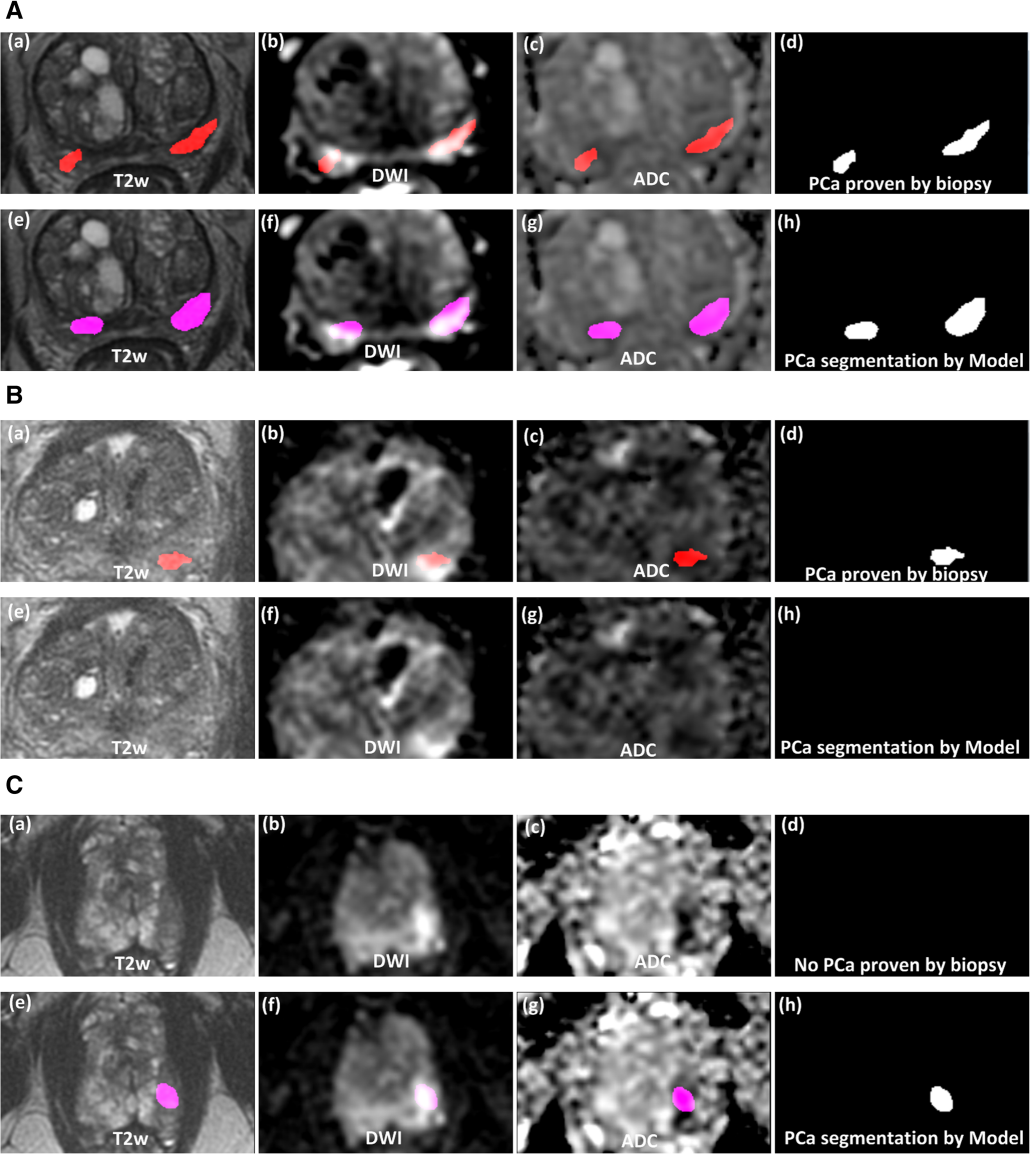
(**A**) Example of a true positive case (age = 75; PSA = 7.5; prostate volume = 61 cc; PIRADS = 3 (left) and 5 (right); ISUP grade = 5 (left) and 3 (right)). The top row shows in overlay the ground truth (in red) as delineated by the radiologist and proven by targeted biopsy as significant PCa. The lower row shows the segmented significant PCa lesion (in pink) by the model. (**B**) Example of a false negative case (age = 69, PSA = 4.5, prostate volume = 47 cc, PIRADS = 4, ISUP grade = 2). The top row shows in overlay the ground truth (in red) as delineated by the radiologist and proven by targeted biopsy as significant PCa. The lower row shows that no PCa lesion was segmented by the model. (**C**) Example of a false positive case (age = 69, PSA = 4.1, prostate volume = 48 cc, PIRADS = 4, ISUP grade = 1). The top row shows no ground truth, the region delineated by the radiologist (not shown) proved by targeted biopsy as insignificant PCa. The lower row shows the false segmented significant PCa lesion (in pink) by the model. This matched the radiologist delineation. All images show the same axial slice as 2D view of mpMRI images (**a**, **e** T2w images; **b**, **f** DWI b800; **c**, **g** ADC map) of the prostate with the reference ground truth (**d**) and the segmented false PCa lesion by model (**h**)

To illustrate the performance of the method visually, three examples (a true positive, a false negative, and a false positive) of PCa segmentation are shown Fig. 6(A–C). In the true positive example (Fig. 6), the model successfully segmented the large and the small lesions as delineated by the radiologist and proven by targeted biopsy as significant PCa. In some cases, PCa segmentation was unsuccessful leading to a false negative (Fig. 6B).In the false positive example (Fig. 6C), the model segments a lesion in the peripheral zone that matches with the radiologist’s delineation; however, the targeted biopsy found no significant PCa.

## Discussion

The use of mpMRI has increased in the early diagnosis of PCa because of its ability to identify suspicious lesions for image-guided biopsy. The MRI-targeted biopsies can improve PCa detection as compared to the random TRUS biopsies [4, 27]. However, to exploit the full benefits of the MRI pathway in the PCa diagnostic process, it is important to increase work efficiency and optimization of the mpMRI analysis, resulting in reduction of under- and overdiagnosis. Optimization of the diagnostic and monitoring process is particularly necessary in low-risk patients on active surveillance, where fear of undergrading is present. An objective qualification and quantification of suspicious lesions on mpMRI may have a positive influence on the monitoring protocol and the (redundant) number of repeated biopsies. Therefore, an automatic approach in monitoring MRI suspicious lesions over time in low-risk patients on active surveillance is indispensable.

In this study, a computer-aided method based on deep learning convolutional neural network to identify PCa in patients on active surveillance was presented. The method used mpMRI (T2w, DWI, ADC map) to segment the PCa with ISUP grade ≥ 2. The performance of the method was evaluated by calculating sensitivity, specificity, and AUC in the total prostate. The average sensitivity achieved by the method was 82–92% at the average specificity of 43–76% by considering different lesion volumes ranging from > 0.03 to > 0.5 cc. The AUC for the average models varied from 0.65 to 0.89. The results showed that the large lesions (> 0.5 cc) can be relatively easily detected and segmented as compared to the smallest lesion volume threshold (≥ 0.03 cc).

In literature, different computer-aided methods are presented to localize PCa [4, 7, 8, 13]. The database used in these studies mostly contained patients, who underwent radical prostatectomy (i.e., high grade and large tumor sizes). Therefore, the usage and advantage of these developed methods is limited in active surveillance population, as these methods cannot deal with the daily reading difficulties of low-risk and small-size PCa. Algohary et al [13] showed that radiomics features from bi-parametric MRI (T2w and ADC map) could accurately detect clinically significant PCa in an active surveillance cohort. However, a limited number of patients (*n* = 56) were included. Furthermore, patients with lesions assigned to PI-RADS suspicion score 3 and with lesions of volume size ≤ 0.5 cc were excluded from the study. The authors showed in two different patients groups that 80% of the positive cases correctly identified as having clinically significant PCa and that 60% of the negative cases were correctly identified as not having clinically significant PCa. In our proposed method, we achieved a higher average sensitivity of 92% at a specificity of 76% by including this subgroup (Fig. 5).

Our study has some limitations. First, our model was specifically trained on an active surveillance cohort; therefore, the results on other patient cohorts (e.g., cohorts of initial diagnosis) may be different. Second, we had access to 116 positive cases, sufficient for algorithm development; however, an increment in the number of training data may improve results. Third, in our data, most of the patient’s PCa lesions (81%) have ISUP grade = 2 (Gleason score = 3 + 4, where 3 represent the most predominant pattern in the biopsy). During training, the network learned features from the dominant non-significant part of the PCa (Gleason score 3) and will segment it in the test data, most particularly in the discordance group 3, which led to a limited specificity. By providing more patient data of high-grade PCa (ISUP grade ≥ 3), the number of false positive segmentations might decrease. Furthermore, the reference ground truth is limited by two factors. First, the accuracy of the MRI-Ultrasound fusion technique (Koelis UroStation™) is reported to range from 3.8 to 5.6 mm [28], with a mean of 4.5 mm. Second, the mean needle placement error is reported to be 2.1 mm [29]. The average combined error will therefore be in the range of 5 mm (0.13 cc). This could affect the localization accuracy of the reference ground truth, and may also influence the results as can be seen for the lower volume thresholds (Fig. 5a, b).

Implementing the proposed method in daily clinical routine has the potential to improve the diagnostic accuracy and monitoring process of prostate cancer. The proposed method can be utilized as second reading, confirming, adding, modifying, or even changing the original decision. Furthermore, the automatic identification and segmentation of the lesions during surveillance will provide consistent quantitative analysis over time, alerting significant changes in volume or conspicuity. The eventual real value will need to be established in prospective clinical use.

## Conclusion

This study presents a deep learning–based computer-aided diagnostic method with acceptable diagnostic accuracy to identify and segment significant (ISUP grade ≥ 2) prostate cancer in patients on active surveillance. The evaluation of the method showed that an average sensitivity of 92% can be achieved with specificity of 76% at the lesion volume threshold > 0.5 cc. The proposed deep learning computer-aided method yields promising results in the automatic identification and segmentation of significant (ISUP grade ≥ 2) prostate cancer in low-risk patients. Low-risk patients may benefit from this objective qualification and quantification of MR images by computer-aided methods, since MRI readings are most difficult in low-volume and low-grade tumors.

## Electronic supplementary material

ESM 1 (DOCX 23 kb)

## Abbreviations

AUC: Area under the curve
CNN: Convolution neural network
DRE: Digital rectal examination
HIPAA: Health Insurance Portability and Accountability Act
ISUP: International Society of Urological Pathology
MRI: Magnetic resonance imaging
PCa: Prostate cancer
PI-RADS: Prostate Imaging Reporting and Data System
PZ: Peripheral zone
ROC: Receiver operating characteristic
TRUS: Transrectal ultrasound
TZ: Transition zone
US: Ultrasound
