# Supplementary material for: Clinically significant prostate cancer detection and segmentation in low-risk patients using a convolutional neural network on multi-parametric MRI
Source: Eur Radiol. 2020 Jun 27;30(12):6582–92. doi: 10.1007/s00330-020-07008-z (PMC7599141; doi:10.1007/s00330-020-07008-z)
Supplement: Supplementary file 1 — (DOCX 23 kb) [file 330_2020_7008_MOESM1_ESM.docx]

**SUPPLEMENTARY MATERIALS**

***Analyses between transition zone and peripheral zone lesions.***

A sub-cohort analysis between transitional zone and. peripheral zone lesions was done by calculating Lesion Segmentation Fraction (LSF) in both zones separately. Where

$$LSF_{PZ}= \frac{True positive segmented lesions in PZ}{Total lesions in PZ}$$

$$LSF_{TZ}= \frac{True positive segmented lesions in TZ}{Total lesions in TZ}$$

LSF were calculated for three lesion volumes >0.03 cc, >0.1 cc and >0.5 cc and results are shown in Table S1 and S2.

Table S1: Lesion segmentation fraction for peripheral zone lesions.

| **Model** | **Total Lesion in test Images** | **LSF at different Lesion volumes** | | |
| --- | --- | --- | --- | --- |
|  |  | **> 0.03 cc** | **> 0.1 cc** | **> 0.5 cc** |
| 1 | 41 (Set 3) | ${33}/{41}=0.80$ | ${31}/{38}=0.82$ | ${11}/{12}=0.92$ |
| 2 | 35 (Set 1) | ${27}/{35}=0.77$ | ${26}/{30}=0.87$ | ${11}/{12}=0.92$ |
| 3 | 36 (Set 2) | ${27}/{36}=0.75$ | ${26}/{33}=0.79$ | ${13}/{13}=1.00$ |
| **Model 1-3** | **112 (Set 1 + Set 2 + Set 3)** | **87/112 = 0.78** | **84/101 = 0.82** | **36/37 = 0.95** |

Table S2: Lesion segmentation fraction for transition zone lesions.

| **Model** | **Total Lesion in test Images** | **LSF at different Lesion volumes** | | |
| --- | --- | --- | --- | --- |
|  |  | **> 0.03 cc** | **> 0.1 cc** | **> 0.5 cc** |
| 1 | 6 (Set 3) | $4/6=0.67$ | $4/6=0.67$ | $3/4=0.75$ |
| 2 | 7 (Set 1) | $7/7=1.00$ | $6/6=1.00$ | $3/3=1.00$ |
| 3 | 10 (Set 2) | $9/{10}=0.90$ | $9/9=1.00$ | $7/7=1.00$ |
| **Model 1-3** | **23 (Set 1 + Set 2 + Set 3)** | **20/23 = 0.87** | **19/21 = 0.90** | **14/14 = 0.93** |

***MRI Acquisition Protocol:***

The mpMRI was acquired using a protocol defined in PI-RADS v2 guidelines. The acquisition parameters are shown in Table S3.

Table S3: Description of mpMRI parameters.

| **Sequence** | **Coil** | **TR** | **TE** | **BW** | **FOV** | **FA**  **degrees** | **SL (mm)** | **Spacing**  **(mm)** |
| --- | --- | --- | --- | --- | --- | --- | --- | --- |
| **T2 FSE-XL Sagittal** | phased-array | 8941 | 140 | 50.00 | 19 | 125 | 3 | 0 |
| **T2 FSE-XL Axial** | phased-array | 5664 | 140 | 62.50 | 19 | 125 | 3 | 0.3 |
| **DWI SE EPI Axial b50,400,800** | phased-array | 3203 | - | - | 24 | 125 | 3 | 0.3 |

FSE-XL, fast spin echo  accelerate; SE EPI, spin echo echo-planer Imaging; TR, time to repetition; TE, time to echo; BW, bandwidth, FOV, field of view; FA, flip angle; SL, slice thickness; DWI, diffusion weighted imaging
